# Supplementary material for: Can Corrole Dimers Be Good Photosensitizers to Kill Bacteria?
Source: Microorganisms. 2022 Jun 7;10(6):1167. doi: 10.3390/microorganisms10061167 (PMC9230841; doi:10.3390/microorganisms10061167)
Supplement: Supplementary file 1 [file microorganisms-10-01167-s001.zip › microorganisms-1733331-supplementary-for update.pdf]

Can corrole dimers be good photosensitizers to kill bacteria?

Paula S. S. Lacerda[1,2], Maria Bartolomeu[1,3], Ana T. P. C. Gomes[4], Ana S. Duarte[4], Adelaide Almeida[1,3], Maria A. F. Faustino[2], Maria G. P. M. S. Neves[2], Joana F. B. Barata[1,*]

[1] CESAM, University of Aveiro, 3810-193 Aveiro, Portugal; placerda@ua.pt (P.S.S.L.); maria.bartolomeu@ua.pt (M.B.); aalmeida@ua.pt (A.A.)

[2] LAQV-REQUIMTE and Department of Chemistry, University of Aveiro, 3810-193 Aveiro, Portugal; faustino@ua.pt (M.A.F.F.); gneves@ua.pt (M.G.P.M.S.N.)

[3] Department of Biology, University of Aveiro, 3810-193 Aveiro, Portugal;

[4] Universidade Católica Portuguesa, Faculty of Dental Medicine (FMD), Center for Interdisciplinary Research in Health (CIIS), 3504-505 Viseu, Portugal; apgomes@ucp.pt (A.T.P.C.G.); asduarte@ucp.pt (A.S.D.)

* Correspondence: jbarata@ua.pt

Table of contents

Materials and Methods content

S1. General information

S2. General procedure for the synthesis of corrole dimers **2 – 4**

S3. Spectrophotometric and spectrofluorimetric measurements

S4. Determination of singlet oxygen production

S5. Determination of DMSO toxicity in *S. aureus* cells

Table S1. Photophysical and photochemical properties of corrole **1** and dimers **2-4** in DMF.

Figure S1. Formation of corrole dimer **S2** in acidic hydrolysis conditions.

Figure S2. Toxicity evaluation of the solvent used (DMSO) in *S. aureus* cells.

Figure S3. Individual values of light and dark controls performed for each PS.

Materials and Methods content

S1. General information

All chemicals and solvents of analytical grade were purchased from Sigma-Aldrich or Merck and used as received.

Analytical Thin Layer Chromatography (TLC) and purification of the reaction mixtures were carried out on pre-coated sheets with silica gel (0.2 mm thick, Merck).

NMR spectra were recorded on Bruker Avance 300 (300.13 MHz for ^{1}H and 282.38 MHz for ^{19}F) and 500 (500.13 MHz for ^{1}H and 125.76 MHz for ^{13}C) spectrometers. $CDCl_3$ (with or without a drop of deuterated pyridine) was used as solvent and TMS (δ = 0 ppm) as the internal standard; the chemical shifts (δ) are expressed in ppm *vs* TMS, and the coupling constants (J) are given in Hz. ^{13}C spectra of compound **4** was improved by internal projection of corresponding HSQC and HMBC experiments.

The ESI-MS spectra were recorded on a linear ion trap mass spectrometer LXQ (ThermoFinnigan, San Jose, CA), instrument. Used conditions were as follows: electrospray voltage was 5 kV in positive mode; the capillary temperature was 275 °C and the sheath gas flow was 5 units. Data acquisition and analysis were performed using the Xcalibur Data System (version 2.0, ThermoFinnigan, San Jose, CA).

S2. General procedure for the synthesis of corrole dimers ***2** – **4***

In a closed reaction tube, corrole **1** (10 mg, 0.01 mmol) was dissolved in the mixture of AcOH (0.24 mL), TFA (0.12 mL) and 5% aq H_2SO_4 (0.06 mL) (4:2:1). The solution was protected from light and stirred at 100 °C under a nitrogen atmosphere. After, the TLC analysis revealed the consumption of the starting corrole **1** and the formation of several new green products. The reaction mixture was cooled to room temperature and carefully neutralized with $NaHCO_3$ (aq). The organic compounds were extracted with CH_2Cl_2, dried over anhydrous Na_2SO_4 and the solvent evaporated under reduced pressure. Then, the resulting residue was purified by TLC using as eluent the mixture hexane/ethyl acetate/pyridine, 156:46:1. Under these conditions, it was possible to isolate two main green fractions with very close R_f and a brownish green fraction with smaller R_f. The less polar fraction was identified as corresponding to the new dimeric structure **4** (14%, higher R_f) followed by known dimer **3** (5%) being the more polar fraction obtained constituted by dimer **2** (15%). Similar procedures were preformed for different acids

combinations. In **Table 1** are presented the reaction conditions (acids used, temperature and reaction time) and the obtained yields for compounds **2**-**4**.

Dimer **2** (experimental data are in agreement with the literature data [1]: ^{1}H NMR ($CDCl_3$, 300.13 MHz): δ 9.43 (s, 4H, H-3,7,3′,17′), 8.74 and 8.55 (2d, 2 × 4H, *J* = 4.8 Hz, 4H, H-7,13,7′,13′ and H-8,12,8′,12′). ^{13}C NMR ($CDCl_3$ + pyridine-d_5, 125.76 MHz): δ 147.4, 144.5, 142.1, 138.2, 134.3, 130.4, 127.7, 124.7, 121.1. ^{19}F NMR ($CDCl_3$, 282.38 MHz): -133.97 (dd, 8F, *J* = 24.8 and 7.9 Hz, *ortho*-F), -134.21 (dd, 4F, *J* = 24.3 and 8.0 Hz, *ortho*-F), -149.48 (t, 4F, *J* = 20.8 Hz, *para*-F), -150.08 (t, 2F, *J* = 20.7 Hz, *para*-F), between -158.09 and -158.53 (m, 8F, *meta*-F), between -158.63 and -158.03 (m, 4F, *meta*-F). UV-Vis (DMF): λ_{max} (relative intensity) 403 (86%), 437 (65%), 620 (11%), 656 (19%), 724 (100%) nm. ESI-MS: *m*/*z* 1722.0 [M - 2py]$^+$.

Dimer **3** (experimental data are in agreement with the literature data) [2]: ^{1}H NMR ($CDCl_3$ + pyridine-d_5, 300.13 MHz): δ 9.29 (d, 2H, *J* = 4.2, H-18,18′), 9.21 and 9.20 (2s, 2 × 1H, H-2 and 2′), 8.92-8.90 (m, 4H, 2H-β + H-17,17′), 8.75 (d, 2H, *J* = 4.8 Hz, H-β), 8.67 and 8.63 (2d, 2 × 2H, *J* = 4.8, H-β). ^{13}C NMR ($CDCl_3$ + pyridine-d_5, 125.76 MHz): δ 127.7 (C-β), 127.6 (C-β), 125.6 (C-β), 124.2 (C-β), 124.1 (C-β), 119.9 (C-2,2′), 117.8 (C-18,18′). ^{19}F NMR ($CDCl_3$, 282.38 MHz): -132.03 (dd, 2F, *J* = 26.4 and 7.8 Hz, *ortho*-F), between -133.93 and -134.16 (m, 8F, *ortho*-F), -134.43 (dd, 2F, *J* = 24.7 and 8.5 Hz, *ortho*-F), - 150.08 (t, 2F, *J* = 21.0 Hz, *para*-F), -150.35 (t, 2F, *J* = 21.1 Hz, *para*-F), -152.49 (t, 2F, *J* = 21.0 Hz, *para*-F), between -158.59 and -159.24 (m, 8F, *meta*-F), between -160.24 and -160.41 (m, 2F, *meta*-F), between -162.34 and -162.50 (m, 2F, *meta*-F). UV-Vis (DMF): λ_{max} (relative intensity) 426 (100%), 583 (13%), 620 (24%) nm. ESI-MS: *m*/*z* 1724.9 [M + H - 2py]$^+$.

Dimer **4**: ^{1}H NMR ($CDCl_3$ + pyridine-d_5, 300.13 MHz): δ 9.72 (s, 1H, H-2), 9.34 (d, 1H, *J* = 3.9 Hz, H-18), 8.94 (d, 1H, *J* = 4.7 Hz, H-β), 8.92 (d, 1H, *J* = 4.5 Hz, H-β), 8.90 (d, 1H, *J* = 3.9 Hz, H-17), 8.85 (d, 1H, *J* = 4.5 Hz, H-β), 8.75 (d, 1H, *J* = 4.5 Hz, H-β), 8.72-8.69 (m, 4H, H-3′ and 3 H-β), 8.65 (d, 1H, *J* = 4.5 Hz, H-β), 8.63 (d, 1H, *J* = 4.5 Hz, H-β), 8.58 (d, 1H, *J* = 4.1 Hz, H-β). ^{13}C NMR ($CDCl_3$ + pyridine-d_5, 125.76 MHz): δ 127.6, (C-β) 127.3 (C-β), 125.3 (C-β), 124.4 (C-β), 124.1 (C-β), 121.1 (C-2), 119.2 (C-β), 118.3 (C-β). ^{19}F NMR ($CDCl_3$, 282.38 MHz): between -133.20 and -134.76 (m, 12F, *ortho*-F), between -149.99 and -150.39 (m, 5F, *para*-F), between -152.04 and -152.27 (m, 1F, *para*-F), between -158.63 and -159.05 (m, 10F, *meta*-F), between -161.04 and -161.33 (br signal, 1F, *meta*-F), between -162.48 and -162.82 (br signal, 1F, *meta*-F). UV-Vis (DMSO): λ_{max} (log ε) 425 (2.69), 608 (1.75). ESI-MS: *m*/*z* 1724.9 [M + H - 2py]$^+$.

S3. Spectrophotometric and spectrofluorimetric measurements

Ultraviolet-visible spectra were recorded under normal air conditions at room temperature with a Shimadzu UV-2501PC spectrophotometer using *N,N*-dimethylformamide (DMF) or dimethylsulfoxide (DMSO), as conveniently referred, in the 350 – 800 nm range and in 1 x 1 cm path length quartz optical cuvette.

Fluorescence emission spectra were recorded on a computer-controlled spectrofluorometer FluoroMax Plus - Horiba Scientific, under normal air conditions at room temperature using DMF as solvent (1 x 1 cm path length quartz cuvette) and in the 500–800 nm range. The fluorescence quantum yields (Φ_{Flu}) of corrole **1** and dimers **2-4** in DMF were determined by comparation of the area under the corrected emission spectrum of each compound with de corresponding of 5,10,15,20-tetraphenylporphyrin (TPP), used as reference (Φ_{Flu} = 0.11 in DMF) [3]. The optical density of each compound and reference was 0.03-0.05 at 420 nm, which was the λ_{exc} used for this determination.

S4. Determination of the singlet oxygen production

The efficacy of corrole monomer **1** and the dimers **2**-**4** to generate singlet oxygen ($^1O_2$) was evaluated using 9,10-dimethylantracene (DMA) as $^1O_2$ scavenger and TPP as reference.

For this, stock solutions of each PS, TPP and DMA (50 mM), were prepared in DMF. In a quartz cuvette, a DMF solution containing each dimer and TPP at an $Abs_{420} \sim 0.10$ was mixed with DMA (30 μM) and irradiated with blue light of 420 nm; all the assays occurred under gentle magnetic stirring at room temperature and in the open atmosphere [4]. The DMA photo-oxidation reaction was monitored by following the absorption at 378 nm for 10 min at 1 min intervals. The DMA photo-oxidation reaction follows a first order kinetics, indicative of $^1O_2$ production. The photostability of DMA in the absence of PS was also monitored under the same irradiation conditions. The results were registered in a first-order plot and obtained as an average of two independent experiments. The singlet oxygen quantum yield (Φ_Δ) was determined using the following equation:

$$\Phi_\Delta = \Phi_\Delta^{std} \frac{K_{sample}}{K_{std}} \frac{1-10^{-Abs_{std}}}{1-10^{-Abs_{sample}}} \quad (1)$$

where Φ_Δ^{std} is the Φ_Δ of TPP used as reference (Φ_Δ = 0.65) [3], K_{sample} and K_{std} are the slope of DMA decay in presence of the PS and TPP, respectively; Abs_{sample} and Abs_{std} are the absorbance of the PS and TPP solutions at 420 nm, respectively.

S5. Determination of DMSO toxicity in S. aureus cells

The tocixity tests of DMSO towards *S. aureus* were performed in a culture medium broth using successive dilutions of the solvent in order to determine its minimal inhibitory concentration (MIC). To do this, a *S. aureus* bacterial suspension at the 0.5 McFarland turbidity standard in Tryptic Soy broth (TSB) fresh medium was prepared from an overnight bacterial culture. Then, the obtained suspension (100 μL) was distributed to each sample-well in a 96-well plate. In the first well it was added a volume of 100 μL of the solvent DMSO and the mixture was homogeneized. A volume of 100 μL was transferred from the first well to the next and two-fold serial dilutions were performed, successively, with a range of DMSO content from 50 to 0.2%. Additionally, bacterial controls were performed, without the addition of DMSO. The 96-well plate was incubated at 37 °C for 24 h, and the optical density (at 600 nm) of the samples was measured with a 96 microplate absorbance reader (Multiskan FC, Thermo Scientific) before and after the 24 h incubation.

Table S1. Photophysical and photochemical properties of corrole **1** and corrole dimers **2-4** in DMF.

compounds	λ_{max} (nm)	λ_{em} (nm)	Stoke's shift (nm)	Φ_{Flu} [a] (±5%)	Φ_{Δ} [b] (±5%)
**2**	403, 438, 476, 656, 723	730	7	0.11	0.22
**3**	426, 585 (sh), 621	661	40	0.11	0.29
**4**	425, 611	656	45	0.09	0.21
**1**	400 (sh), 421, 571, 594	600, 655	6	0.21 [c]	0.69

[a] Using TPP in DMF as reference (Φ_{Flu} = 0.11) [3];

[b] Using TPP in DMF as reference (Φ_{Δ} = 0.65) [3];

[c] This paper and [5].

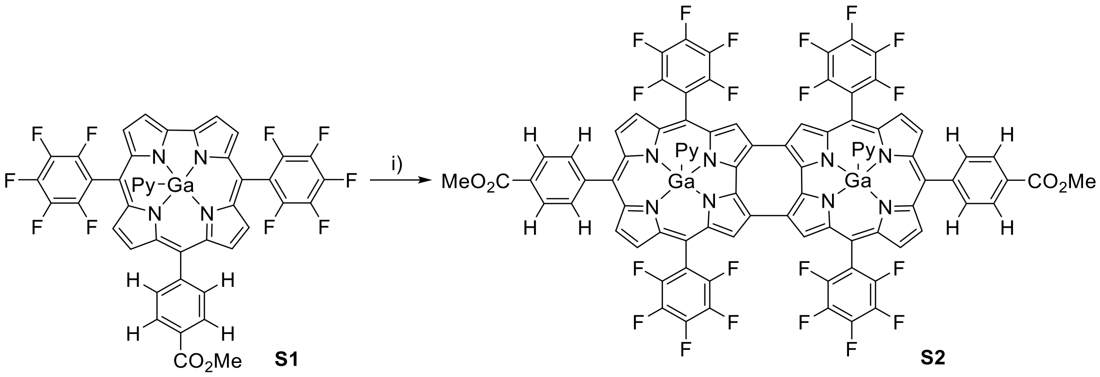

Figure S1. Formation of corrole dimer **S2** in acidic hydrolysis conditions. i) acetic acid + trifluoro acetic acid + 5 % aq sulfuric acid (4:2:1 volume ratio).

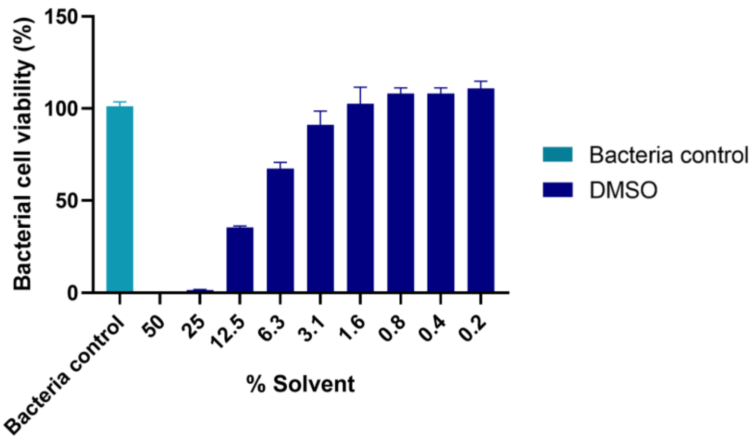

Figure S2. Toxicity evaluation of the solvent used (DMSO) in *S. aureus* cells. According to stock solution concentration of the PS (500 μM), the correspondence between solvent % of 50.0, 25.0, 12.5, 6.3, 3.1, 1.6, 0.8, 0.4, and 0.2% and PS solution concentration is, respectively, 250, 125, 62.5, 31.3, 15.6, 7.8, 3.9, 2.0, and 1.0 μM.

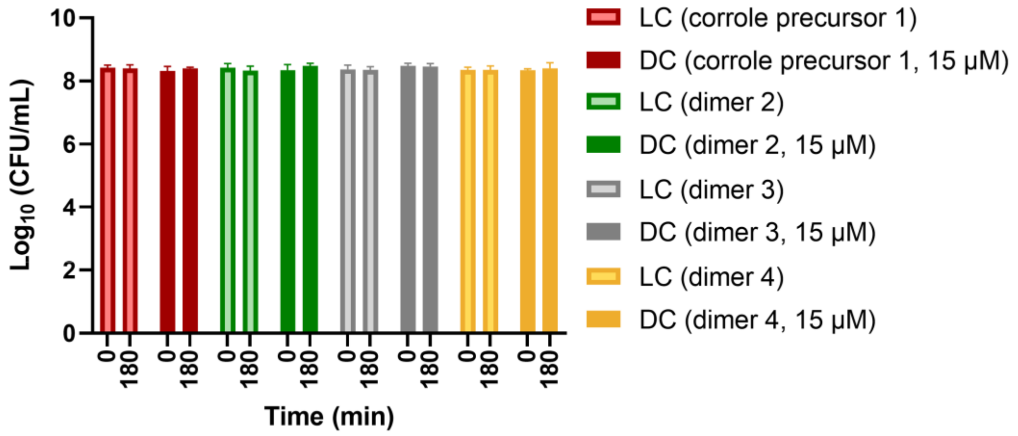

Figure S3. Individual values of light and dark controls performed for each PS. These LC and DC were performed alongside antimicrobial photodynamic therapy. Data values represent the mean of three independent experiments. To make the data easier to read, it is shown only the initial value (0 min) and the final time (180 min) for each set of values.

Bibliography

1. Bhowmik, S.; Kosa, M.; Mizrahi, A.; Fridman, N.; Saphier, M.; Stanger, A.; Gross, Z. The Planar Cyclooctatetraene Bridge in Bis-Metallic Macrocycles: Isolating or Conjugating? *Inorg. Chem.* **2017**, *56*, 2287–2296, doi:10.1021/acs.inorgchem.6b02944.

2. Barata, J.F.B.; Neves, M.G.P.M.S.; Tomé, A.C.; Faustino, M.A.F.; Silva, A.M.S.; Cavaleiro, J.A.S. How light affects 5,10,15-tris(pentafluorophenyl)corrole. *Tetrahedron Lett.* **2010**, *51*, 1537–1540, doi:10.1016/J.TETLET.2010.01.033.

3. Barata, J.F.B.; Zamarrón, A.; Neves, M.G.P.M.S.; Faustino, M.A.F.; Tomé, A.C.; Cavaleiro, J.A.S.; Röder, B.; Juarranz, Á.; Sanz-Rodríguez, F. Photodynamic effects induced by meso-tris(pentafluorophenyl)corrole and its cyclodextrin conjugates on cytoskeletal components of HeLa cells. *Eur. J. Med. Chem.* **2015**, *92*, 135–144, doi:10.1016/J.EJMECH.2014.12.025.

4. Santos, I.; Gamelas, S.R.D.; Vieira, C.; Faustino, M.A.F.; Tomé, J.P.C.; Almeida, A.; Gomes, A.T.P.C.; Lourenço, L.M.O. Pyrazole-pyridinium porphyrins and chlorins as powerful photosensitizers for photoinactivation of planktonic and biofilm forms of E. coli. *Dye. Pigment.* **2021**, *193*, 109557, doi:10.1016/j.dyepig.2021.109557.

5. Wang, L.L.; Wang, H.; Cheng, F.; Liang, Z.H.; Liu, C.F.; Li, Y.; Wang, W.Q.; Peng, S.H.; Wang, X.; Ying, X.; et al. Investigation of excited-state photophysical properties of water-soluble gallium corrole. *J. Phys. Chem. C* **2017**, *121*, 12350–12357, doi:10.1021/acs.jpcc.7b00168.
